# An intelligent film recommender system based on emotional analysis

**DOI:** 10.7717/peerj-cs.1243

**Published:** 2023-03-09

**Authors:** Wenzuixiong Xiong, Yichao Zhang

**Affiliations:** 1 School of art, Hubei University, Wuhan, Hubei, China; 2 National Financing Guarantee Fund, Beijing, Beijing, China

**Keywords:** Film review, PAD emotional model, PSO, Movie recommendation, Micro-blog

## Abstract

The existing personalized film recommendation methods take the user’s historical rating as an important basis for recommendation. However, the user’s rating standards are different, so it is difficult to mine the user’s real preferences and form accurate push. Therefore, to achieve high-quality personalized recommendation of films, it is particularly important to mine the emotion of user reviews. In this article, a personalized recommendation method based on sentiment analysis of film reviews is proposed, where natural language processing technology is used to mine the emotional tendency of user reviews. The multi-modal emotional features are weighted and the weighted fusion feature vector after PSO is taken as the overall emotion vector, then the emotional similarity of weighted fusion is calculated by considering the time factor of content publishing and the average emotional tendency of users. By calculating the matching degree of emotional value between users and films, the top-N film recommendation for target users is given. The test results show that the effect of the personalized film recommendation system based on multimodality is superior to that of the comparison method, which effectively solves the problem of different user rating scales, and really increases users’ interest in watching films.

## Introduction

The Internet film community website app platforms generate a large number of film reviews, which provides an important reference for the majority of potential audience groups to make film viewing choices. At the same time, netizens and film lovers have gradually developed a consumption feedback mode of sharing the experience of watching films on Internet-related platforms. These feedback forms the text reviews of film review platform in the form of text, providing a large amount of emotional analysis data for research, which is of great significance in the field of emotional analysis in film review.

Network information is quite large, but due to the lack of user personal data the supply and demand of information is asymmetric, which makes it more difficult for people to get personalized recommendation. Recommendation system is an important method to solve information overload and avoid information island. Therefore, recommendation system is one of the most successful methods in machine learning systems  (Quadrana, Cremonesi & Jannach, 2018). For example, the tertiary industry represented by Internet + services has become an important factor in the growth of national economy. In the traditional recommendation method, user rating is regarded as an important index to judge the user’s tendency. It assumes that users with similar scores have similar preferences, while neighboring users can not completely objectively and truly reflect their own preferences (Hou, Li & Wang, 2017). To some extent, user rating data can represent the user’s attitude towards the product, but the reason for the difference in user rating cannot be reasonably explained, and the comment is thought in the mind, which can better reflect the user’s psychology. Moreover, psychological research shows that most people have herd mentality, and people’s preference or emotional state of goods will be affected by the majority of people’s emotions (Mingxing, 2016). Therefore, in order to achieve accurate recommendation, mining the emotion of user comments becomes particularly important.

One of the most important personalized recommendation algorithms used in current social networks is collaborative filtering recommendation algorithm. Collaborative filtering algorithm uses user rating information to construct user rating matrix, and completes recommendation by calculating the similarity between users. However, it also has some defects, such as the algorithm only uses the user’s rating information, but ignores other information of the user for the project, including evaluation time, comment content, etc. (Zhang, Qiang & Duan, 2021). For example, when a user wants to know about a film, in addition to viewing the average score of the film, the quality of the popular film reviews of the film is also the aspect of the user’s inspection. The review information of other users can provide reference opinions on whether to choose to watch a film. In addition, the emotional information contained in user comments also reflects the potential characteristics of the project itself. In addition to scoring, the expression of users’ preferences for items in social networks also plays an important role in the text review information of projects (Bandara et al., 2021). When users comment on the items they like, their emotions tend to be positive, while for the items they don’t like, the comments will produce negative emotions (Su et al., 2019). Therefore, in the process of discovering users’ interests and preferences, their comments on the project play an important role. However, users’ emotional preferences are not just binary opposites, which can be more expressed as happiness, trust, gratitude, praise, love, accident, anger, derogation, *etc.* (Eerola & Vuoskoski, 2011). It is meaningful to find user preferences and improve the quality of user recommendation service by using comment vector representation and multi granularity sentiment analysis. Meanwhile, a user’s preference for the same event is not consistent, but changes with the influence of external events or decreases over time.

To enhance the effect of personalized film recommendation and realize the differentiated recommendation based on user emotion, this article tries to solve the problem of emotional modeling between users and films, the emotional feature fusion of image and text, and the similarity calculation based on emotion. By introducing the idea of PSO optimization, multi-modal emotion features are weighted and fused, and the feature vector weighted and fused by particle swarm optimization is taken as the overall emotion vector, and the emotional similarity of weighted fusion is calculated by considering the factors of the release time of user Weibo content and the average emotional tendency of users.

## Related Works

### Emotional computing

The key of text-based emotion computing is to accurately understand natural language based on the analysis of the syntax and semantic structure of text sentences. Turney (2007) analyzed and calculated the similarity degree of words with the intention of introducing point mutual information, and reconstructed the composition of the dictionary by introducing polarity semantics, which increased the richness of part of speech structure in the dictionary. Chen & Yang (2013), based on the pre-set emotion dictionary, introduced LDA model tool to further extract the subject words in the dictionary, expanded the richness of emotional tendency, and provided more sufficient supplement for polysemy subject words of a word. Zhou, Yang & Yang (2013) introduced information entropy to identify the Chinese text information. The innovation lies in the use of the SO-PMI algorithm to further screen the emotional subject words in the text, and construct a new emotion dictionary for the new subject words. Li, Cao & Cao (2008) used the weight first extraction method to extract the head word, and integrated the traditional machine learning method with the weight priority to obtain the semantic trend of the text.

Affective computing from other modes (such as images and videos) has only recently begun to be considered (Bakshi, Kaur & Kaur, 2016). The purpose of multimedia emotion computing is to identify the audience’s emotion expected to be caused by a given stimulus (Zhao et al., 2017). One of the main challenges of multimedia emotion computing is the emotional gap, that is, the inconsistency between the features and the expected emotional state of users brought by perceptual signals (Hanjalic, 2006; Zhang, 2019). Perceptual inconsistencies make it insufficient to simply predict the major (average) emotion categories of highly subjective variables. Zhao et al. (2016) proposed that two multimodal emotional computing tasks can be performed to deal with subjective challenges: predicting the personalized emotional perception of each viewer and assigning multiple emotional tags to each stimulus. For the latter task, the multi label learning method can be used to assign multiple tags to each stimulus of equal importance, or predict the emotional distribution and try to learn the degree of each emotion (Yang, Sun & Sun, 2017).

The ambiguity and ambiguity of the semantic ambiguity of natural language and the multi-scale nature of the text content, especially the interaction of sarcasm, metaphor, politeness, linguistic and cultural characteristics and style, result in the critical technical challenge of accurately describing and defining the hybrid subdivision emotion. Most of the existing methods do not consider the timeliness of the user’s emotional attribute tags, so it is difficult to describe the dynamic emotional attributes of users.

### Film recommendation

Collaborative filtering algorithm is the most commonly used film recommendation algorithm, but it relies on user historical data and has the problem of sparse data cold start. Therefore, many scholars have improved it. Jia & Yu (2019) proposed a modified score collaborative filtering algorithm, which modified the user score matrix through the confidence in user clustering information and association rule mining, and used the modified score method to replace the weighted calculation score algorithm, hence working on the exhibition of the recommendation algorithm. Yu et al. (2019) proposed a collaborative filtering algorithm to improve similarity. Considering whether the film has been seen or not, a collaborative filtering recommendation algorithm combining Pearson correlation coefficient and Jaccard algorithm is proposed with full consideration of the user scoring situation. Chen et al. (2018) thought that the existing recommendation algorithms, including shallow and deep methods, usually embed the user’s history into a single potential vector, which may have lost the correlation between the user’s history and future interest by item or feature level. In addition, Bayes, clustering, Horting, SVD and machine learning  (Breitfuss et al., 2021; Cintia Ganesha Putri, Leu & Seda, 2020) and other algorithms have also been applied to the recommendation system. Töscher, Jahrer & Bell (2009) proposed a hybrid recommendation algorithm, which combined different learning algorithms to achieve better accuracy. Bell & Koren (2007) improved the cosine measurement method in collaborative filtering algorithm, and used statistical user ratings to compare user rating data, thus enhancing the recommendation effect of collaborative filtering.

## Emotional Analysis Model of Film Reviews

### User emotion description model

**Figure 1:**
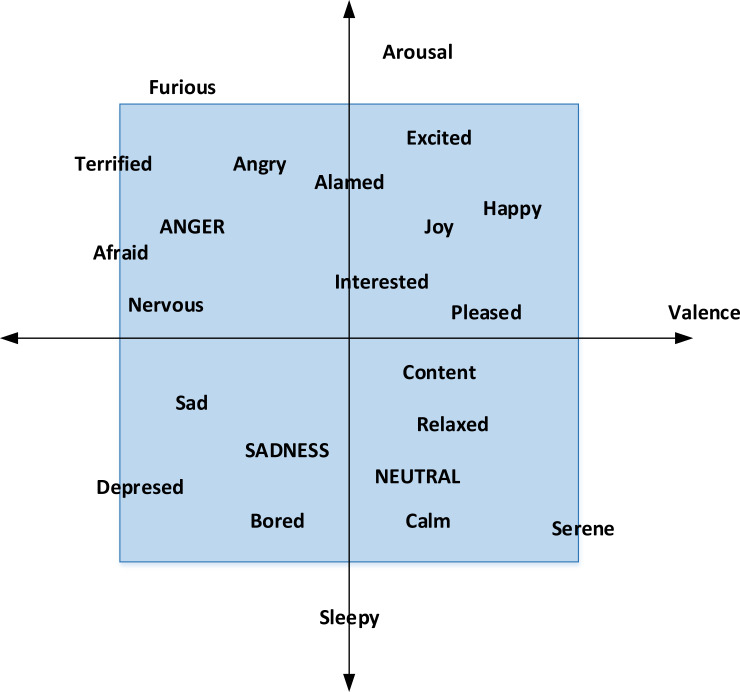
PAD emotion classification.

The PAD three-dimensional emotional model is selected for user emotion modeling. Dimension emotion theory points out that emotion should be described in psychological dimension space. The theory benefits from expressing various emotions that are not necessarily described by specific emotion descriptors. As far as affective computing tasks are concerned, it is also a problem of the accuracy of discrete emotion classification, while dimensional emotion is the regression problem of continuous space. The emotional classification of film recommendation is shown in Fig. 1. The vertical axis is the activation dimension, which represents the intensity of emotion, and the horizontal axis is the quantity of potency and an evaluation of the degree of positive and negative emotions.

**Figure 2:**
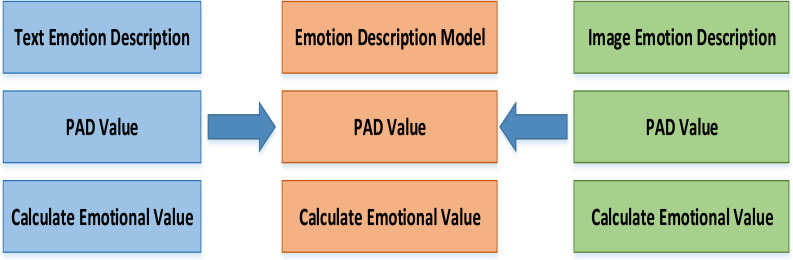
Emotion expression model.

The emotion description model designed in this article is shown in Fig. 2. It defines the major categories of emotion description model. The subclass includes text emotion description and image emotion description, which can inherit the function of the parent class and add its own new functions.

 Different symbols can be used to describe the user and alphanumerics can be used to enhance the display. Social media is the window of human emotion. A user’s historical data from Sina Weibo can be collected, and image and text data can also be extracted from Sina Weibo. These data can then be used to build a user sentiment tendency model.

Comment data contains a lot of image and text information, so the user-defined model based on comment data is constructed by combining text and image. For user u, we collect all original tweets, including posted photos, to form data set W.

Text and pictures in comment do not contain tag information and must be annotated automatically first. Suppose comment data package contains comment data w and image set ${I}_{u}= \left\{ {i}_{1},{i}_{2},\ldots ,{i}_{n} \right\} $, traverse all users’ comment data and obtain emotion attribute PAD vector.

(1) \begin{eqnarray*}{\text{WeiboSenti}}_{u}={X}_{u}= \left\{ {x}_{u1},{x}_{u2},\ldots ,{x}_{\mathrm{un}} \right\} .\end{eqnarray*}

In addition, film data contains rich pictures and text information, so the movie emotion model based on movie data is established by combining the text and pictures, that is, the emotion tendency value of the movie is calculated. For a movie m, we collect its published data set M containing titles, introductions, reviews, and images. Each element in M can be defined by title, introduction, comment, and corresponding photo set ${I}_{m}= \left\{ {i}_{1},{i}_{2},\ldots ,{i}_{n} \right\} $.

Because the text and pictures of the film do not contain any label information, it is necessary to label them first. For a film, the PAD feature vector of emotion distribution can be obtained through emotion analysis model.

(2) \begin{eqnarray*}{X}_{m}= \left\{ {x}_{m1},{x}_{m2},\ldots ,{x}_{\mathrm{mn}} \right\} .\end{eqnarray*}

### Image-text feature fusion based on PSO

#### Feature construction

$T= \left\{ {t}_{1},{t}_{2},\ldots ,{t}_{p} \right\} $ represents the input text features, *p* represents the dimension of the text attribute vector, $V= \left\{ {v}_{l},{v}_{2},\ldots ,{v}_{q} \right\} $ represents the input image features, and *q* represents the dimension of the image attribute vector. Define the characteristic space $\Omega = \left\{ {\omega }_{1},{\omega }_{2},\ldots ,{\omega }_{c} \right\} $, where *ω*_*k*_ represents a fusion feature, and *X* is a feature cascade set of text feature *T* and image feature *V*.

Firstly, the input text feature *T* and image feature *V* are normalized respectively, and then the two features are spliced according to Formula (3)

(3) \begin{eqnarray*}F=T+A.\end{eqnarray*}

Where the feature dimension of each line of *F* is *p* + *q*, and “+” represents feature series. The problem based on feature fusion is to get the mapping relation of feature set *X*_*j*_ to *ω*_*k*_, denoting *f*:*X*_*j*_ → *ω*_*k*_.

#### Algorithm description

Since values of PAD are continuous real numbers, the problem of predicting text pad values should be regression rather than classification. Therefore, the output of the model cannot use the classification activation function softmax. Instead, using a linear activation function, which can be defined by Formula (4).

(4) \begin{eqnarray*}y={W}_{d}{\mathrm{x}}_{t}+{b}_{d}\end{eqnarray*}

where x_*t*_ is the vector learned in the fusion layer, y is the P or A or D value of the target text (Pleasure-displeasure, Arousal-nonarousal, Dominance-submissiveness), and the weight *W*_*d*_ and bias quantity *b*_*d*_ of the linear decoder. The model iteratively solves the mean square error between the predicted value and the true value of y. If given a set of training samples $\mathrm{X}= \left\{ {\mathrm{x}}^{ \left( 1 \right) },{\mathrm{x}}^{ \left( 2 \right) },\ldots ,{\mathrm{x}}^{ \left( m \right) } \right\} $, and their emotional value set PAD ${V}_{pad}^{{}^{{^{\prime}}}}=\mathrm{y}= \left\{ {\mathrm{y}}^{ \left( 1 \right) },{\mathrm{y}}^{ \left( 2 \right) },\ldots ,{\mathrm{y}}^{ \left( m \right) } \right\} $. Then, the loss function can be calculated by Formula (5).

(5) \begin{eqnarray*}L(\mathrm{x},\mathrm{y})= \frac{1}{2m} \sum _{i=1}^{m}\,{ \left\| h \left( {\mathrm{x}}^{ \left( i \right) } \right) -{y}^{ \left( i \right) } \right\| }^{2}.\end{eqnarray*}

In the training stage, we used the stochastic gradient descent algorithm and the error back propagation algorithm to update the weight parameters of each layer. The error unit of the gradient descent method of linear decoder is changed by Formula (6):

(6) \begin{eqnarray*}{\delta }^{ \left( i \right) }=- \left( {y}^{ \left( i \right) }-f \left( {\mathrm{x}}_{t}^{ \left( l \right) } \right) \right) ={W}_{d}{\mathrm{x}}_{t}^{ \left( i \right) }+{b}_{d}-{y}^{ \left( i \right) }\end{eqnarray*}

where, ${\delta }^{ \left( i \right) }$ is the error element to be corrected backwards. ${\mathrm{x}}_{t}^{ \left( i \right) }$ is the feature vector obtained after the fusion layer of the i-th training sample, ${y}^{ \left( i \right) }$ is the PAD value of the sample marker, f is the function of linear decoder.

Since the fusion weights of regression models of feature fusion lack universality, PSO is introduced to determine the fusion weights of regression models. Firstly, the weights *w*_1_, *w*_2_, *w*_3_, of feature fusion are randomly initialized, and the output of the fusion model is shown in Formula (7).

(7) \begin{eqnarray*}{V}_{pad}={W}_{d} \left( {w}_{1}\cdot A+{w}_{2}\cdot T+{w}_{3}\cdot C \right) +{b}_{d}.\end{eqnarray*}

In the expression, *W*_*d*_ and *b*_*d*_ table show the weight and bias of the linear decoder, respectively. Gradient descent is used to continuously update weight parameters. When the loss function of the model converges to a stable value, the fusion model is stopped and the PSO algorithm is used to learn the fusion weight of features.

After weighted fusion of text and image emotion feature vectors by particle swarm optimization, the fusion vector is mapped to pad emotion space through a full connection layer, and then three-dimensional emotion vector is obtained. The objective optimization value is the minimum value of the difference between the real value and the predicted value, so as to optimize the corresponding weight coefficient of the text emotion feature vector and the text feature vector, and output the emotion vector which can represent the emotion to the greatest extent, so as to support the calculation of the emotion matching degree. In this article, the minimum fitness value is calculated as Formula (8).

(8) \begin{eqnarray*}F=\sum _{1}^{n}\,{ \left\| {V}_{\text{pad}}-{V}_{\text{pad}}^{{}^{{^{\prime}}}} \right\| }^{2}.\end{eqnarray*}

The actual output emotion value *V*_*pad*_, the predicted output emotion value ${V}_{pad}^{{}^{{^{\prime}}}}$, n represents the number of samples.

## Personalized Movie Recommendation based on Emotion Analysis Model of Film Review

The user’s emotion is simulated by emotion computing, and the future emotional state is predicted, and the personalized recommendation with high quality experience is given. Next, describe the emotion computing model, and then introduce the user personalized emotion recommendation method based on it.

### Affective state transition

Affective state transition (AST) refers to the user’s change from one emotional state to another. AST is represented by a directed graph *G*(*AS*, *DE*), where *AS* is the set of emotional states and *DE* is the set of directed edges. *AS*_*i*_ → *AS*_*j*_ represents the AST from *AS*_*i*_ to *AS*_*j*_, and $P \left( A{S}_{i}\rightarrow A{S}_{j} \right) $ represents the probability of the AST from *AS*_*i*_ to *AS*_*j*_. Given a finite emotional state, the AST can be represented by the following matrix, whose element is the probability of the AST $ \left\{ P \left( A{S}_{i}\rightarrow A{S}_{j} \right) \right\} $.

(9) \begin{eqnarray*}P(AS)_{M\times M}= \left( \begin{array}{@{}cccc@{}} \displaystyle A{S}_{1,1}&\displaystyle A{S}_{1,2}&\displaystyle \cdots &\displaystyle A{S}_{1,M}\\ \displaystyle A{S}_{2,1}&\displaystyle A{S}_{2,2}&\displaystyle \cdots &\displaystyle A{S}_{2,M}\\ \displaystyle \vdots &\displaystyle \vdots &\displaystyle \ddots &\displaystyle \vdots \\ \displaystyle A{S}_{M,1}&\displaystyle A{S}_{M,2}&\displaystyle \cdots &\displaystyle A{S}_{M,M} \end{array} \right) \end{eqnarray*}

where ${\mathop{\sum }\nolimits }_{j=1}^{M}A{S}_{i,j}=1,$i =1 , …, *M*; *AS*_*i*,*j*_ ≠ *AS*_*j*,*i*_. In the task of this article, 8 × 8 AST matrix $P(AS)_{8\times 8}= \left\{ A{S}_{i,j}\mid 1\leq i,j\leq 8 \right\} $, where 0 ≤ *ES*_*i*,*j*_ ≤ 1. The AST prior probability $P \left( A{S}_{i,j} \right) $ can be initialized, which represents the probability of self-loops with the same starting and ending emotional states. Emotional recommendation items identified from the user’s history list of comment.

Emotion is a complex physiological reaction of human beings, which is limited by many complex factors, so it is very difficult to predict the dynamic emotion of users. For the convenience of the study, the following hypothesis is given: according to the list of users’ comment history, the change of users’ emotional state is most likely to be caused. Given a history list of user u ${W}_{u}= \left\{ {w}_{1},{w}_{2},\ldots ,{w}_{N} \right\} $, the corresponding emotion sequence is represented by PAD emotion model $A{S}^{u}= \left\{ A{S}_{1}^{u},A{S}_{2}^{u},\ldots ,A{S}_{N}^{u} \right\} $, emotional sequence has influence on the excitation of continuous emotional state $A{S}_{N+i}^{u}$. According to the model, the influence of each comment in list *W*_*u*_ on current emotional state can be calculated.

The discriminant model directly learns the decision function f(x), that is, it models the mapping from input space to output space. Or directly to the distribution P (Y — X) model, namely the case markers in feature X Y appear probability, is the a posteriori probability. The discriminant and conditional probability theory are used to predict the dynamic emotional states. Under the condition of the observation value *AS*^*u*^, the probability of the user’s current possible emotional states is calculated. Therefore, the influence probability of each emotional state in *AS*^*u*^ is given.

(10) \begin{eqnarray*}P \left( A{S}_{j+k}^{u}\mid A{S}_{j}^{u} \right) ,1\leq j\leq N,1\leq j+k\leq N,\sum _{j=1}^{N}\,P \left( A{S}^{u}\mid A{S}_{j}^{u} \right) =1.\end{eqnarray*}

### Calculation of emotion matching degree

**Figure 3:**
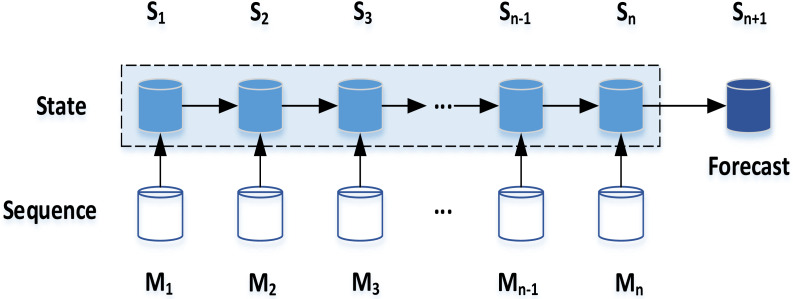
User emotional state prediction model.

Conditional Random Field (CRF) is the basic model of natural language processing. According to the comment history list, the CRF in Fig. 3 is used to predict the user’s emotional state.

The proposed scheme in this article is based on the prediction of users’ current emotional state. The emotional sequence obtained from users’ comment history list can not only predict users’ current emotional state, but also divide it into sub-sequences based on the same emotional state. Given a list of comment ${W}_{u}= \left\{ {w}_{1},{w}_{2},\ldots ,{w}_{N} \right\} $, *k* emotional states can be obtained through emotion recognition, then the sub-sequence represented by ${W}_{u} \left( A{S}_{i} \right) $ is selected as the recommendation basis, and the optimization ranking recommendation list with the highest emotion matching degree with ${W}_{u} \left( A{S}_{i} \right) $ is output.

According to the performance of emotion recognition, emotion features are output and mapped into PAD emotion space to form specific emotion representation. Emotional value of PAD can be represented as *pad* = *A*^*T*^ℌ. Therefore, the emotion matching degree can be calculated by the following inner sum.

(11) \begin{eqnarray*}\mathfrak{F} \left( {w}_{ui},{m}_{c} \right) = \left( {A}_{ui}^{\mathrm{T}}\mathfrak{H} \right) { \left( {A}_{c}^{\mathrm{T}}\mathfrak{H} \right) }^{\mathrm{T}}={A}_{ui}^{\mathrm{T}}\mathfrak{H}{\mathfrak{H}}^{\mathrm{T}}{A}_{c}\end{eqnarray*}

where, *w*_*ui*_ represents the emotional state of the predicted user, and *m*_*c*_ represents the emotional state of the *c*-th movie to be recommended. Given *N* recommended items, their emotional matching degree can be expressed by emotional similarity matrix, where each item ${F}_{k} \left( A{S}_{i},A{S}^{u} \right) $ is non-negative.

## Experiment and Analysis

### Data set

In this article, a cross platform user sentiment preference modeling method is proposed. By extracting the personalized information of users, the overlapping users of the two platforms are modeled. The data of film comments in this article comes from IMDB dataset, and Film data from MovieLens dataset. Using the crawler technology, we obtained 2896 common users of Sina and Douban, downloaded the film list from Douban film website, including film ID, film title, film introduction, actor, director, etc., and the evaluation form includes user ID, film ID, score, comment and time stamp. A total of 8,273 reviews of 1,910 films by 2896 users where each user rates at least 20 movies.

### Comparison of emotion feature recognition effect

In order to verify the effectiveness of the multi-modal fusion method, feature fusion and PSO optimized weighted feature fusion features are applied to PAD emotion recognition. In PSO algorithm, the initial population size is set to 20, and the inertia *n* = 0.5, learning factors 1 and 2 are 8 and 6, respectively

**Figure 4:**
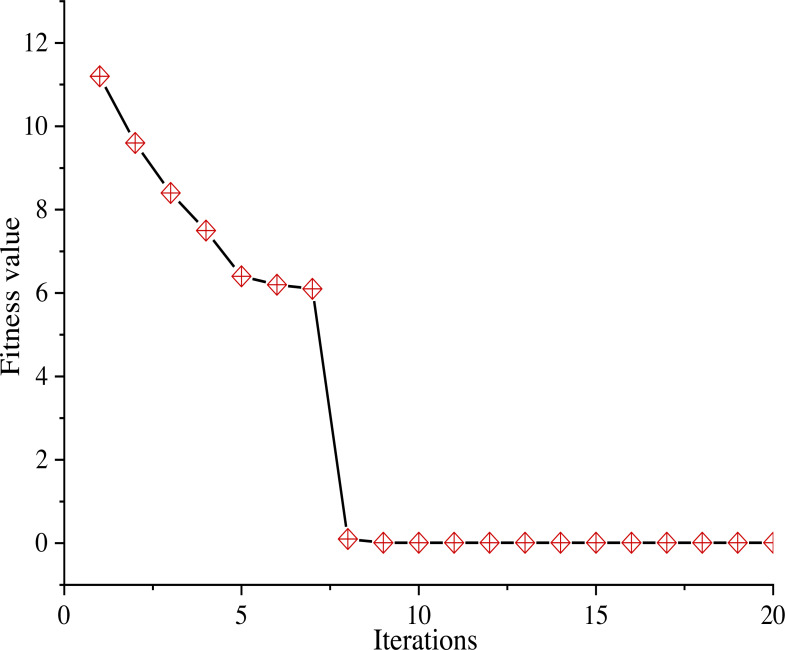
PSO convergence curve.

The feature weighted fusion emotion value recognition uses PSO algorithm iteration to find the optimal parameters, as shown in Fig. 4.

Figure 4 shows that when PSO algorithm learns weights, the best fitness value converges with the increase of iteration times. With the increase of iteration times, the fitness value decreases from the initial value, and tends to converge after eight iterations to obtain the global optimal value.

### Comparison of different algorithms

**Figure 5:**
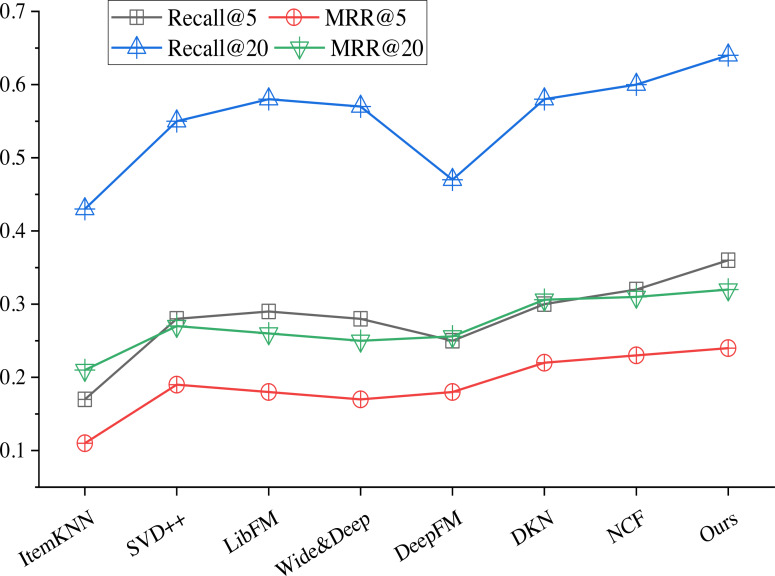
Comparison of different algorithms.

The performance of the proposed algorithm is evaluated by comparing with several baseline methods. The results are shown in Fig. 5. Comparison methods include the following.

ItemKNN algorithm (Sarwar et al., 2001). In this method, the items to be recommended were clustered into several classes, and the average score of each class was used as the algorithm prediction score:

SVD++ algorithm (Koren, 2008). This method introduced implicit feedback on the basis of singular value decomposition (SVD);

Libfm (Rendle, 2012). This is a feature-based matrix decomposition. In the experiment, TF-IDF features are extracted from the items that users click on and candidate items, and the two types of features are connected as the input of LibFM;

Wide & deep (Cheng et al., 2016) used the combination of wide channel of linear transformation;

Deepfm (Guo et al., 2017) is a recommendation method combining decomposer and deep neural network, and the two parts use the same TF-IDF features;

DKN (Wang et al., 2018) learns the items to be recommended and the representation of users, where a set of embedding vectors is obtained for the historical items that users click on, and then the candidate items are automatically matched to each item by attention mechanism, and they are aggregated with different weights.

NCF (He et al., 2017) is a collaborative filtering based on neural network. which uses multi-layer perceptron and takes comment count information as input.

The model presented in this article significantly improves each of the evaluation indicators. Similarly, the improvement in the indicators of Recall@5 and MRR@5 was particularly marked. For the personalized recommendation system, the more advanced the recommendation movie is, the more it can capture the user’s attention. Therefore, the better effects of Recall@5 and MRR@5 reflect the better user satisfaction and experience brought by the personalized recommendation algorithm.

By integrating emotional information into the recommendation model to build a recommendation system, the results verify the feasibility of the proposed recommendation algorithm through multimodal emotion fusion. In terms of reflecting the recall performance of the recommendation system, the algorithm has achieved the best results, which shows that the algorithm can solve the personalized recommendation problem of the recommendation system based on multimodal emotion fusion to a certain extent.

### Recommendation effect evaluation

To evaluate the recommendation results of the proposed film recommendation algorithm, the accuracy of film recommendation is evaluated according to the emotional value by measuring the integrity of the recommendation results and the target film. Through subjective evaluation, the recommendation results of this algorithm are compared with those of random selection method. Each user is randomly provided with the first five recommendations obtained from one of the two methods, and they independently choose the number of films they think match the film they want to watch.

**Figure 6:**
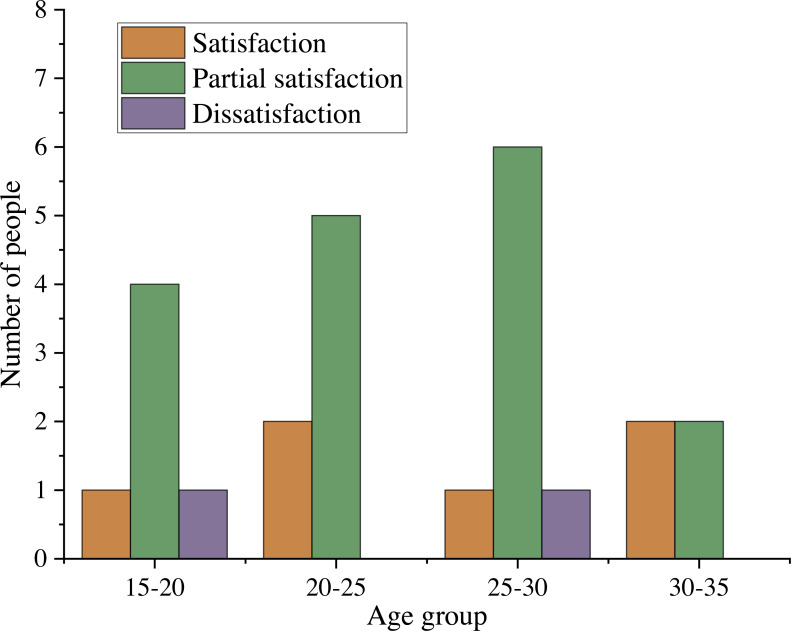
Evaluation of film recommendation effect in different age groups.

The model proposed in this article can extract the emotions of users before watching movies, classify them, and recommend movies that are in line with the current emotions of users. Regardless of whether users are in positive or negative emotions, the successful recommendation accuracy is high. In addition, 25 historical information records of different ages were collected, and five movies were recommended for them to participate in model training (see Fig. 6). It can be seen that only 8% of people do not like the recommended films, indicating the effectiveness of this recommendation method.

## Conclusion

To enhance the effect of film recommendation and realize the differentiated recommendation based on user emotion, this article tries to solve the problem of emotional modeling between users and films, the emotional feature fusion of image and text, and the similarity calculation based on emotion. The experimental results show that the effectiveness of the personalized recommendation system based on multimodality is better than that of the comparative method. Moreover, the research in this article adds explicit and implicit emotional factors on the basis of the original similarity calculation, which further improves the effect of personalized recommendation. Therefore, it has better performance than the original method, which can be well applied to personalized film recommendation, and has good performance in recommendation accuracy and recall rate.

##  Supplemental Information

10.7717/peerj-cs.1243/supp-1 Supplemental Information 1 Code used for the researh

10.7717/peerj-cs.1243/supp-2 Supplemental Information 2 The code compression package

10.7717/peerj-cs.1243/supp-3 Data S1 Data

## Additional Information and Declarations

### Data Availability

The following information was supplied regarding data availability: The data and code are available in the Supplemental Files and the datasets are also available at: - MovieLens Latest Datasets:
https://grouplens.org/datasets/movielens/latest/. - Twitter dataset: https://www.kaggle.com/datasets/davidmartngutirrez/twitter-bots-accounts?resource=download&select=twitter_human_bots_dataset.csv.
