# Supplementary figures and images for: An intelligent film recommender system based on emotional analysis
Source: PeerJ Comput Sci. 2023 Mar 9;9:e1243. doi: 10.7717/peerj-cs.1243 (PMC10280678; doi:10.7717/peerj-cs.1243)

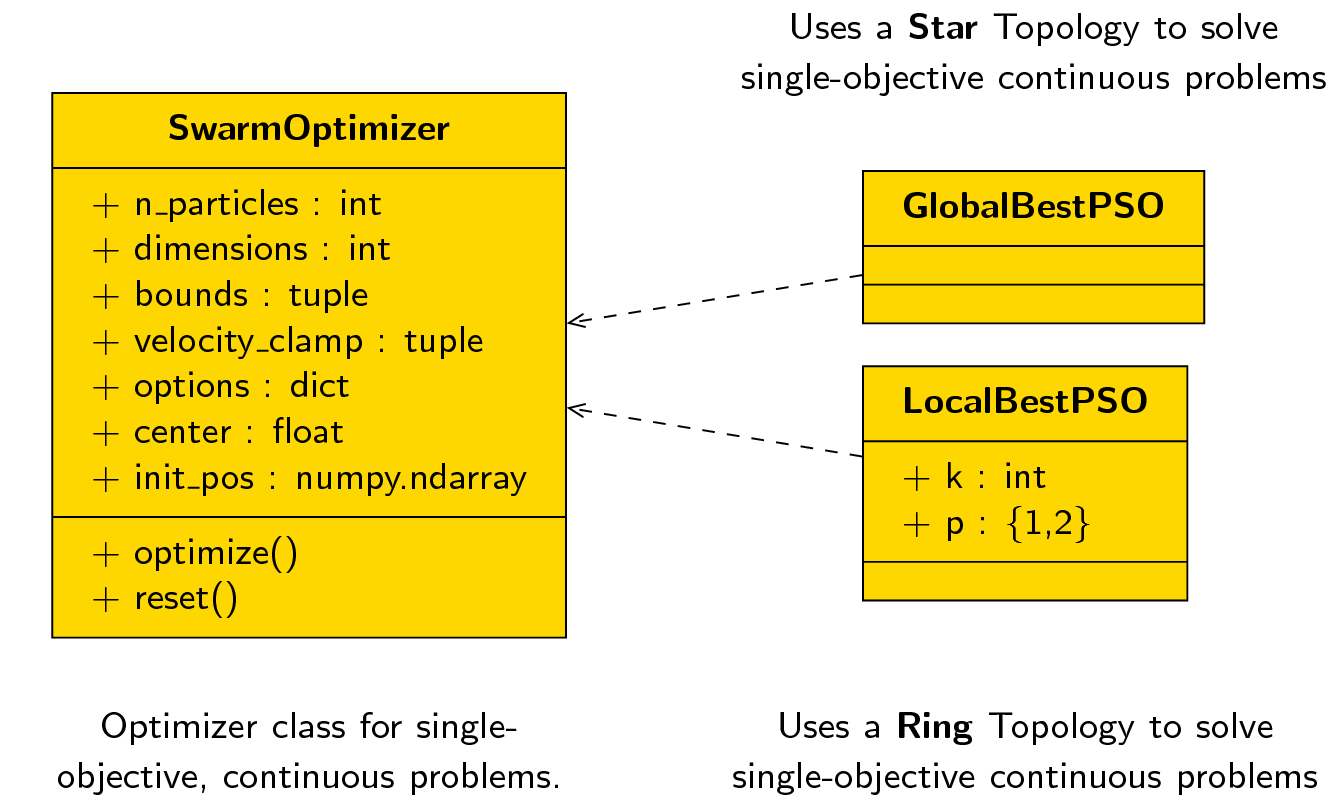

Supplement: Supplemental Information 2 [file peerj-cs-09-1243-s002.zip › ┤·┬δ/data mining/docs/assets/inheritance.png]

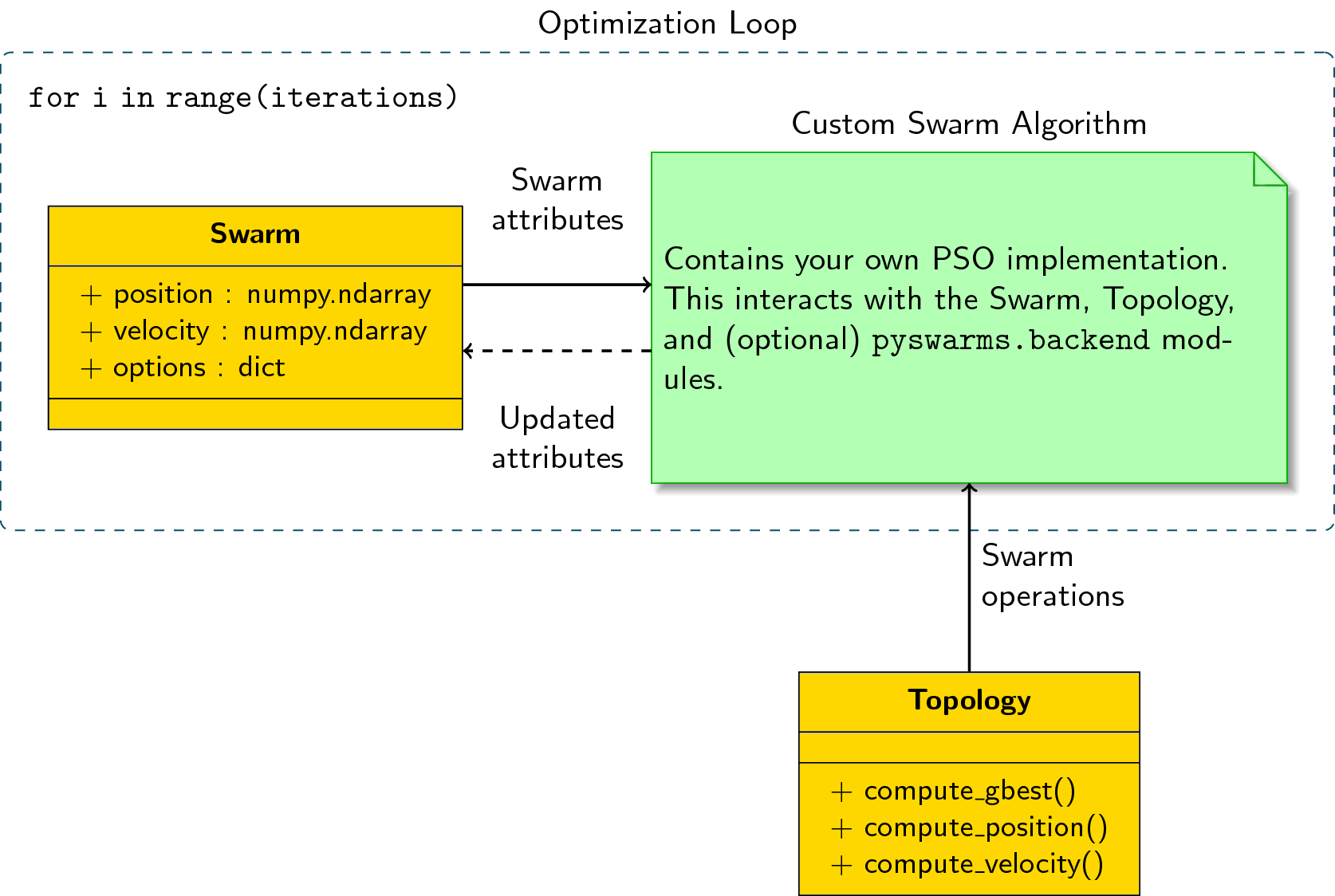

Supplement: Supplemental Information 2 [file peerj-cs-09-1243-s002.zip › ┤·┬δ/data mining/docs/assets/optimization_loop.png]

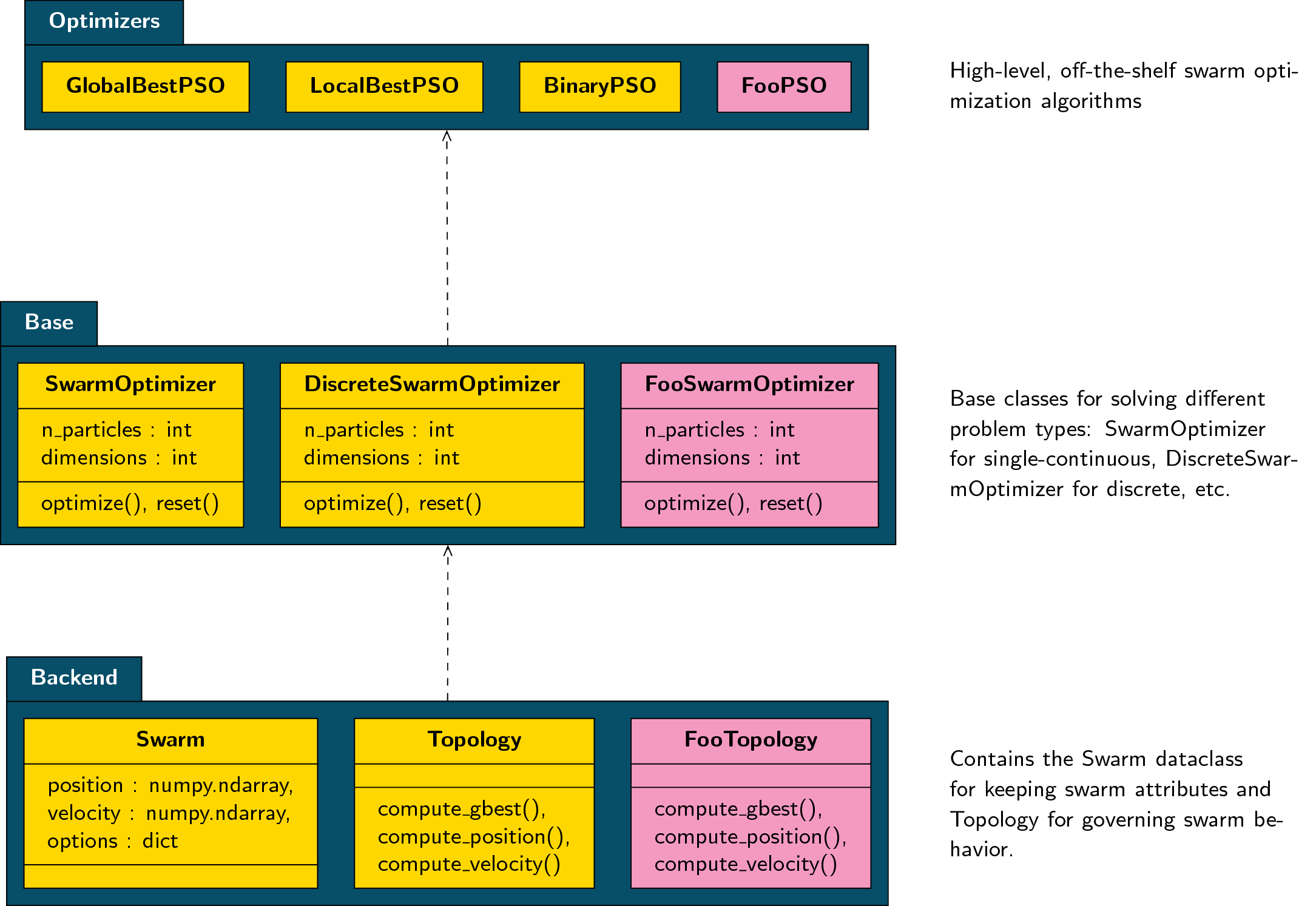

Supplement: Supplemental Information 2 [file peerj-cs-09-1243-s002.zip › ┤·┬δ/data mining/docs/assets/pyswarms_api.png]

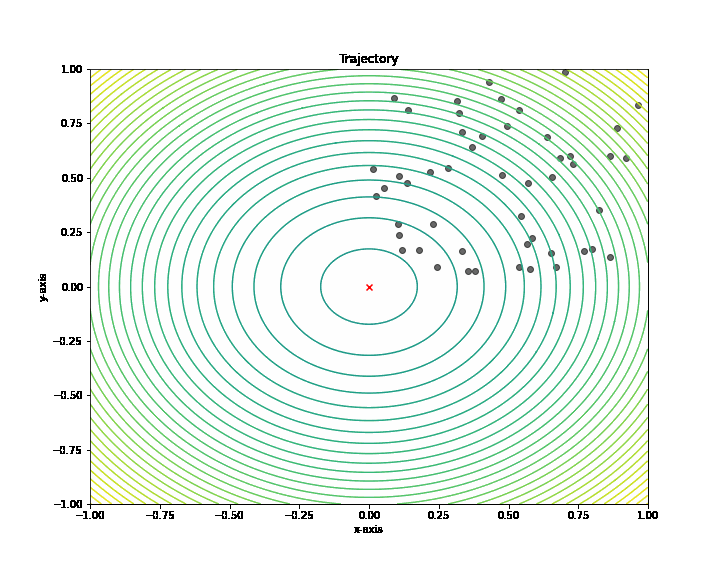

Supplement: Supplemental Information 2 [file peerj-cs-09-1243-s002.zip › ┤·┬δ/data mining/docs/examples/tutorials/ani.gif]

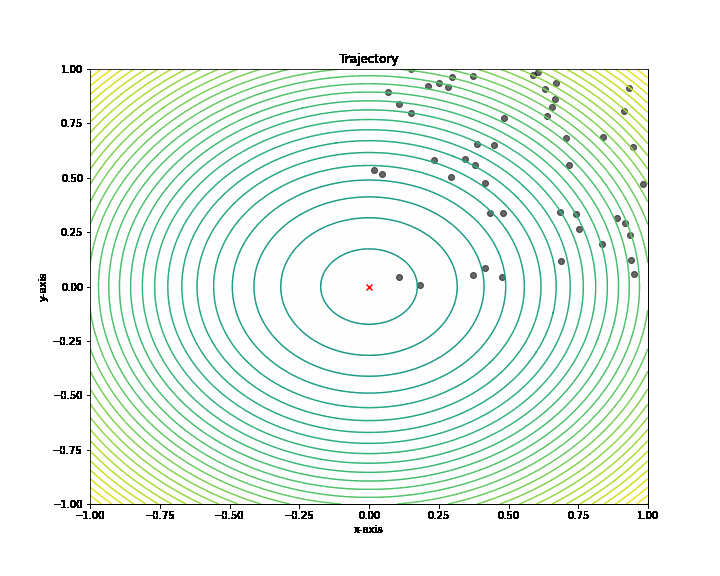

Supplement: Supplemental Information 2 [file peerj-cs-09-1243-s002.zip › ┤·┬δ/data mining/docs/examples/tutorials/ani_h.gif]

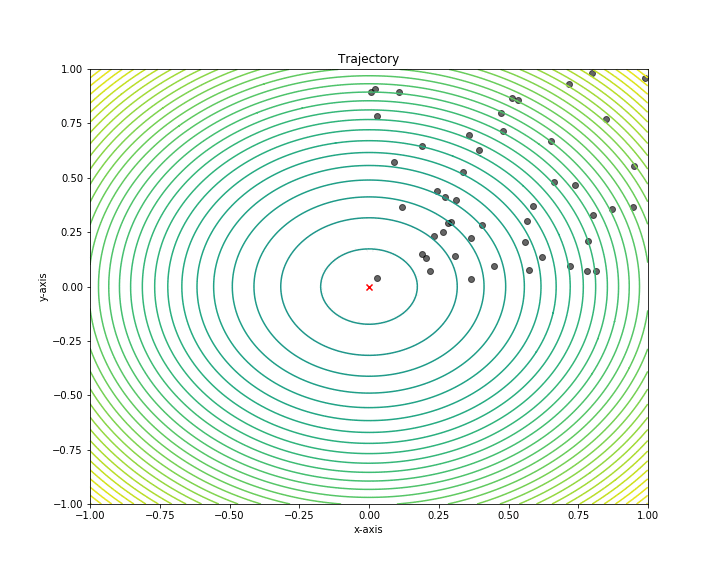

Supplement: Supplemental Information 2 [file peerj-cs-09-1243-s002.zip › ┤·┬δ/data mining/docs/examples/tutorials/plot0.gif]

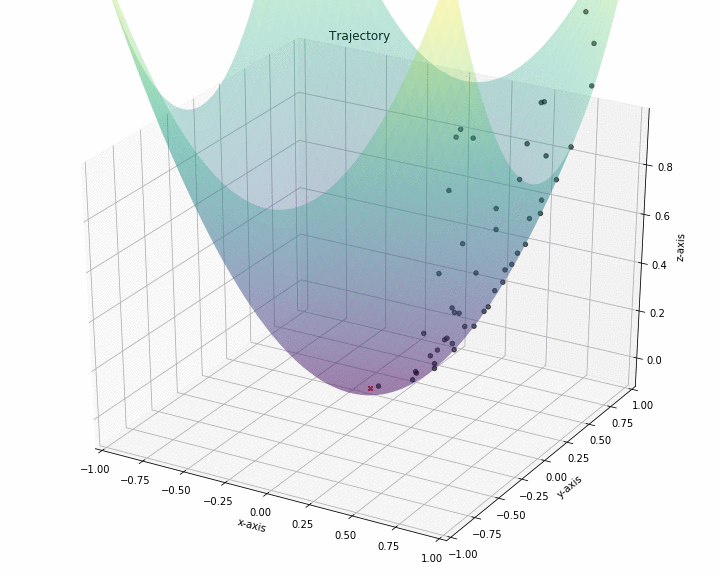

Supplement: Supplemental Information 2 [file peerj-cs-09-1243-s002.zip › ┤·┬δ/data mining/docs/examples/tutorials/plot1.gif]

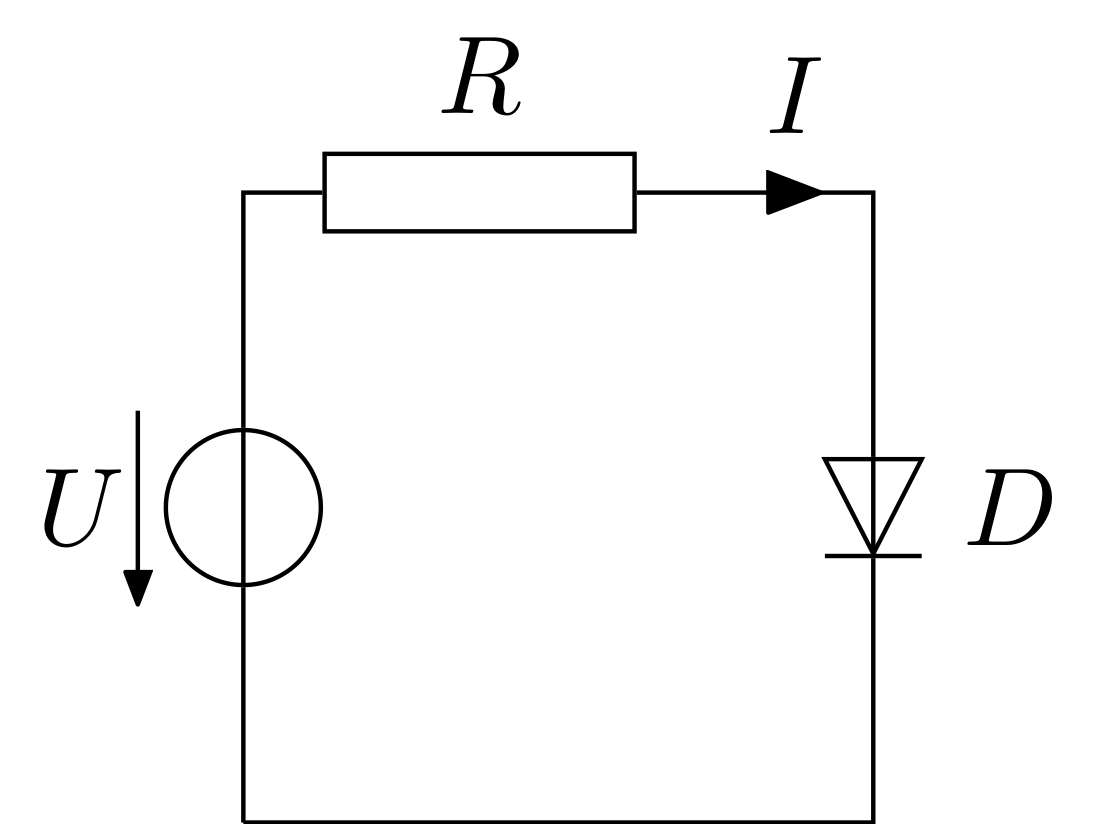

Supplement: Supplemental Information 2 [file peerj-cs-09-1243-s002.zip › ┤·┬δ/data mining/docs/examples/usecases/circuit.png]

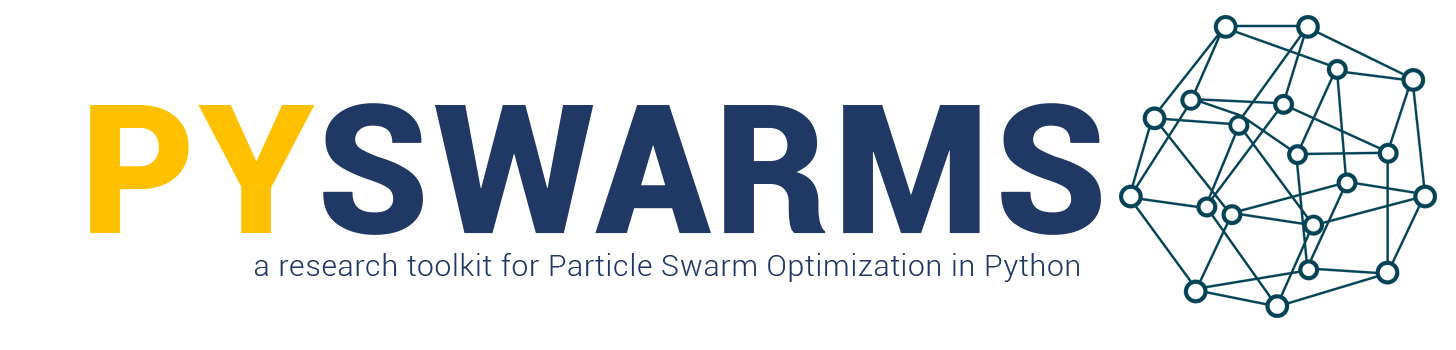

Supplement: Supplemental Information 2 [file peerj-cs-09-1243-s002.zip › ┤·┬δ/data mining/docs/pyswarms-header.png]
